# Photoperiod Regulates Corticosterone Rhythms by Altered Adrenal Sensitivity via Melatonin-Independent Mechanisms in Fischer 344 Rats and C57BL/6J Mice

**DOI:** 10.1371/journal.pone.0039090

**Published:** 2012-06-15

**Authors:** Tsuyoshi Otsuka, Mariko Goto, Misato Kawai, Yuki Togo, Katsuyoshi Sato, Kazuo Katoh, Mitsuhiro Furuse, Shinobu Yasuo

**Affiliations:** 1 Laboratory of Regulation in Metabolism and Behavior, Faculty of Agriculture, Kyushu University, Fukuoka, Japan; 2 Department of Animal Physiology, Faculty of Agriculture, Tohoku University, Sendai, Japan; Nagoya University, Japan

## Abstract

Most species living in temperate zones adapt their physiology and behavior to seasonal changes in the environment by using the photoperiod as a primary cue. The mechanisms underlying photoperiodic regulation of stress-related functions are not well understood. In this study, we analyzed the effects of photoperiod on the hypothalamic-pituitary-adrenal axis in photoperiod-sensitive Fischer 344 rats. We first examined how photoperiod affects diurnal variations in plasma concentrations of adrenocorticotropic hormone (ACTH) and corticosterone. ACTH levels did not exhibit diurnal variations under long- and short-day conditions. On the other hand, corticosterone levels exhibited a clear rhythm under short-day condition with a peak during dark phase. This peak was not observed under long-day condition in which a significant rhythm was not detected. To analyze the mechanisms responsible for the photoperiodic regulation of corticosterone rhythms, ACTH was intraperitoneally injected at the onset of the light or dark phase in dexamethasone-treated rats maintained under long- and short-day conditions. ACTH induced higher corticosterone levels in rats examined at dark onset under short-day condition than those maintained under long-day condition. Next, we asked whether melatonin signals are involved in photoperiodic regulation of corticosterone rhythms, and rats were intraperitoneally injected with melatonin at late afternoon under long-day condition for 3 weeks. However, melatonin injections did not affect the corticosterone rhythms. In addition, photoperiodic changes in the amplitude of corticosterone rhythms were also observed in melatonin-deficient C57BL/6J mice, in which expression profiles of several clock genes and steroidgenesis genes in adrenal gland were modified by the photoperiod. Our data suggest that photoperiod regulates corticosterone rhythms by altered adrenal sensitivity through melatonin-independent mechanisms that may involve the adrenal clock.

## Introduction

Most species living in temperate zones adapt their physiology and behavior to seasonal changes in the environment by using the photoperiod as a primary cue. Mechanisms underlying the photoperiodic regulation of reproduction have been well documented in birds and mammals; recent studies successfully characterized molecular events in brain that trigger photoperiodic responses of gonads in Japanese quail [1]–[3], sheep [4], [5], and mice [6], [7]. On the contrary, a limited number of studies have been reported regarding the mechanisms underlying the photoperiodic regulation of stress-related functions. For example, in Syrian hamsters (*Mesocricetus auratus*), photoperiod modifies plasma concentrations of glucocorticoids (corticosterone and cortisol) [8] as well as their responses to acute stress [9], but the controlling mechanisms remain unclear.

Several studies focused on the role of the negative feedback loop within the hypothalamic-pituitary-adrenal axis (HPA axis) in the photoperiodic regulation of the stress response. In Syrian hamsters, a short photoperiod induces type I glucocorticoid receptor binding in the hippocampus and hypothalamus [9] and mineralocorticoid receptor mRNA expression in the hippocampus [10]. Similarly, in white-footed mice (*Peromyscus leucopus*), a short photoperiod increases corticosterone responses to restraint and glucocorticoid receptor gene expression in the hippocampus with enhanced sensitivity to dexamethasone-mediated suppression of corticosterone [11]. However, the causal relationship between photoperiodic changes in feedback regulation and photoperiod-driven changes in corticosteroid levels is debatable because of the lack of data on ACTH secretion in animals exposed to various photoperiodic stimuli. In addition, most studies examined physiological outputs from the HPA axis (e.g., corticosterone levels, their responses to acute stress, and glucocorticoid/mineralocorticoid receptor expression) in a single time point per day under each photoperiodic condition, despite of the diurnal variations in these outputs in rodents [12]–[15].

In mammals, photoperiodic information perceived by the retina is transmitted to the pineal gland through sympathetic innervations via the suprachiasmatic nucleus (SCN), a master biological clock, and translated into patterns of melatonin secretion that correlate with photoperiod duration [16], [17]. Photoperiodic melatonin signals exert their effects on various physiological functions such as reproduction via melatonin receptors located in the target tissue. This is mainly regulated through the activation of MT1 receptors expressed in the hypophyseal pars tuberalis [18], [19]. Functional MT1 receptors are also distributed in the adrenal gland of rats [20], although their roles in photoperiodic regulation in adrenal physiology remain unclear. In addition to melatonin signals, the photoperiod regulates physiological functions through sympathetic innervations. For example, in Siberian hamsters, sympathetic nerves innervating white adipose tissue contribute to seasonal regulation in adiposity [21], and the sympathoadrenal system is associated with photoperiodic changes in immune function [22]. Thus, the photoperiod coordinates precise physiological outputs through hormonal and neural pathways according to the distribution of receptors and nerve innervations in the target tissue. However, the relative contribution of each pathway remains elusive in organs such as the adrenal gland that have both melatonin receptors and sympathetic nerve innervations. As regards the generation of corticosterone rhythms under 12 h light:12 h dark condition, light exposure regulates circadian clock genes in the adrenal gland via sympathetic nerve innervations to control the corticosterone release [23], and the adrenal clockwork is linked to steroidogenesis by several genes including steroidogenic acute regulatory protein (*StAR*), a gene encoding a rate-limiting enzyme in steroidogenesis [24], [25].

To understand the mechanisms underlying photoperiodic regulation of the HPA axis, we focused on these mechanisms in the Fischer 344 inbred strain of rat. This strain exhibits robust responses to the photoperiod regarding gonadal growth, body weight [26], and expression levels of type 2 deiodinase in the mediobasal hypothalamus [27], a key gene for photoperiodic gonadal regulation [2]. This study further aimed to address the roles of melatonin on photoperiodic regulation of corticosterone rhythms by 1) melatonin injection experiments in rats and 2) analysis of corticosterone rhythms in relation to molecular clockwork and steroidgenesis genes in the adrenal gland of C57BL mice. This mouse strain cannot produce detectable levels of melatonin due to a truncation in arylalkylamine *N*-acetyltransferase [28], [29].

## Results

### Photoperiodic Regulation in Body Weight and Epididymal Fat Mass

**Figure 1:**
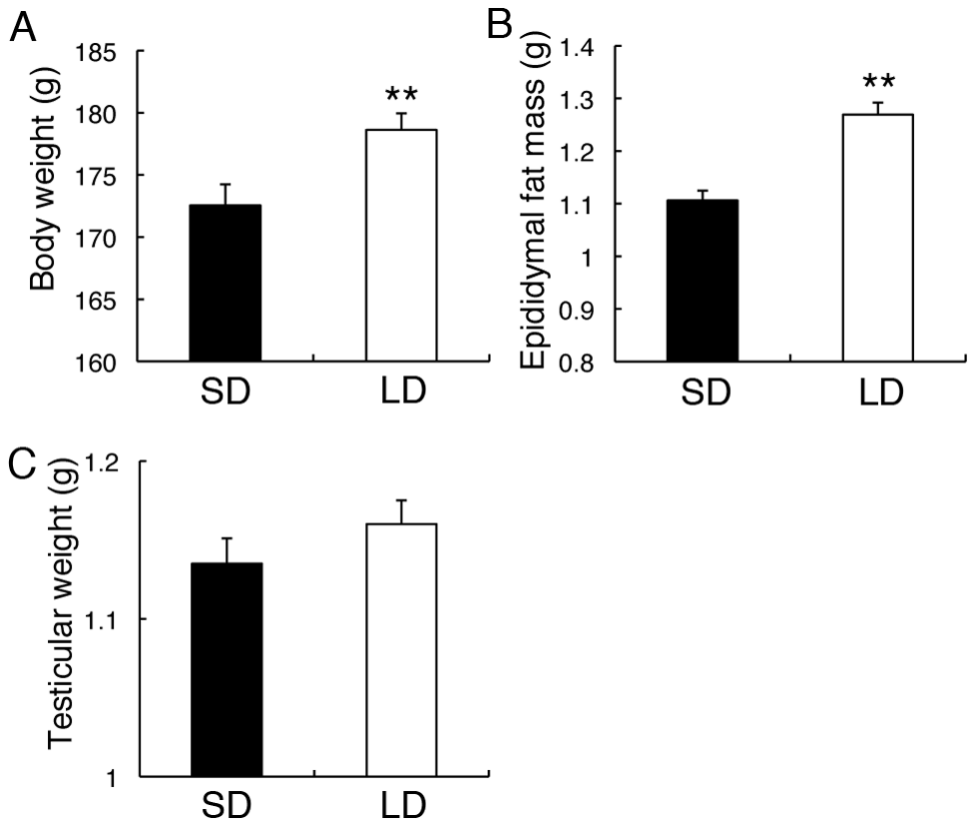
Effects of photoperiod on body weight, epididymal fat mass, and testicular weight in Fischer 344 rats. Rats were maintained under either short-day condition (SD, 8 h of light, 16 h of darkness, *black bars*) or long-day condition (LD, 16 h of light, 8 h of darkness, *white bars*) for 2 weeks. Body weight (A) and epididymal fat mass (B) of the rats maintained under LD were larger than those maintained under SD, whereas testicular weight (C) did not differ between rats maintained under SD and LD. Testicular weight and epididymal fat mass are indicated as the average of left and right specimens. **, *P*<0.01, *t*-test. Values are means + SEM (n=25).

To confirm the photoperiodic response of Fischer 344 rats used in this study, we examined the effects of photoperiod on body weight, epididymal fat mass, and testicular weight in rats maintained under long- and short-day conditions. Body weight and epididymal fat mass of the rats maintained under long-day condition were greater than those in rats maintained under short-day condition (*P*<0.01) (Figure 1A and B). However, testicular weight did not differ between rats maintained under long- and short-day conditions (Figure 1C).

### ACTH and Corticosterone Rhythms in Plasma

**Figure 2:**
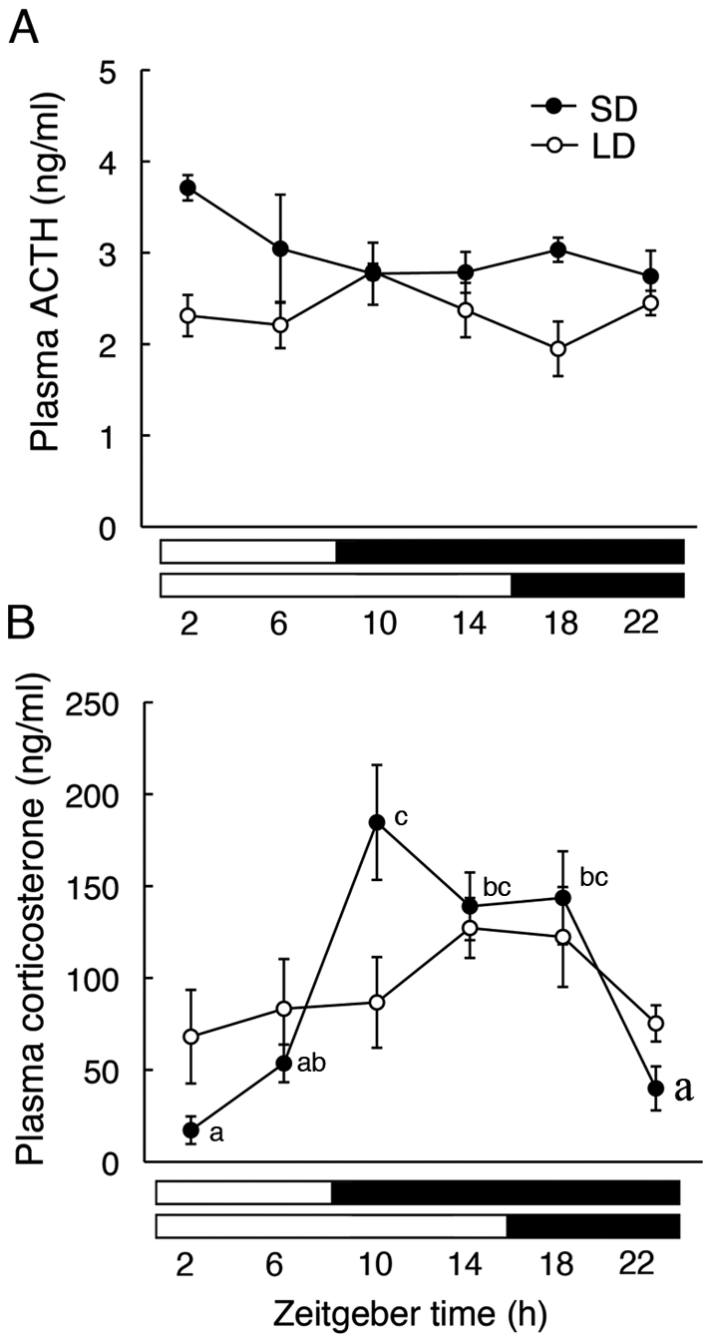
Effects of photoperiod on diurnal fluctuations in plasma ACTH and corticosterone of Fischer 344 rats. Rats were maintained under either short-day condition (SD, 8 h of light, 16 h of darkness, *black circles*) or long-day condition (LD, 16 h of light, 8 h of darkness, *white circles*) for 2 weeks. Corticosterone levels (A) in rats maintained under SD showed a significant variation throughout 24 h (*P*<0.01, one-way ANOVA), whereas no diurnal variation was detected in those maintained under LD. ACTH levels (B) in rats maintained under LD was constitutively lower than those under SD (*P*<0.01, two-way ANOVA). Different characters indicate significant differences among time points within the SD (*P*<0.05, one-way ANOVA followed by Bonferroni's multiple comparison test). The black and white bars below the graphs show the length of the dark and light phases. Values are means ± SEM (n=4–5).

Next, we examined temporal changes in the plasma concentrations of ACTH and corticosterone throughout a 24-h cycle under long- and short-day conditions. ACTH levels exhibited no diurnal variations under both photoperiodic conditions (long-day condition: F_5,17_=1.76; short-day condition: F_5,19_=1.56, *P*>0.05) (Figure 2A). Two-way ANOVA revealed a significant difference in relation to photoperiod (F_1,36_=17.62, *P*<0.01); ACTH levels in animals exposed to long-day condition were constitutively lower than those exposed to short-day condition (Figure 2A). Corticosterone levels exhibited a clear diurnal variation in animals exposed to short-day condition (F_5,19_=13.57, *P*<0.01) with a peak that ranged from Zeitgeber time (ZT: ZT0 corresponds to light onset) 10 to 18, whereas no significant variation was observed in those exposed to long-day condition (F_5,18_=1.28, *P*>0.05) (Figure 2B). Photoperiod had a significant effect on the temporal patterns of corticosterone levels (F_5,36_=3.6, *P*<0.01, interaction between photoperiod and time).

### Effects of Photoperiod on the HPA Axis

**Figure 3:**
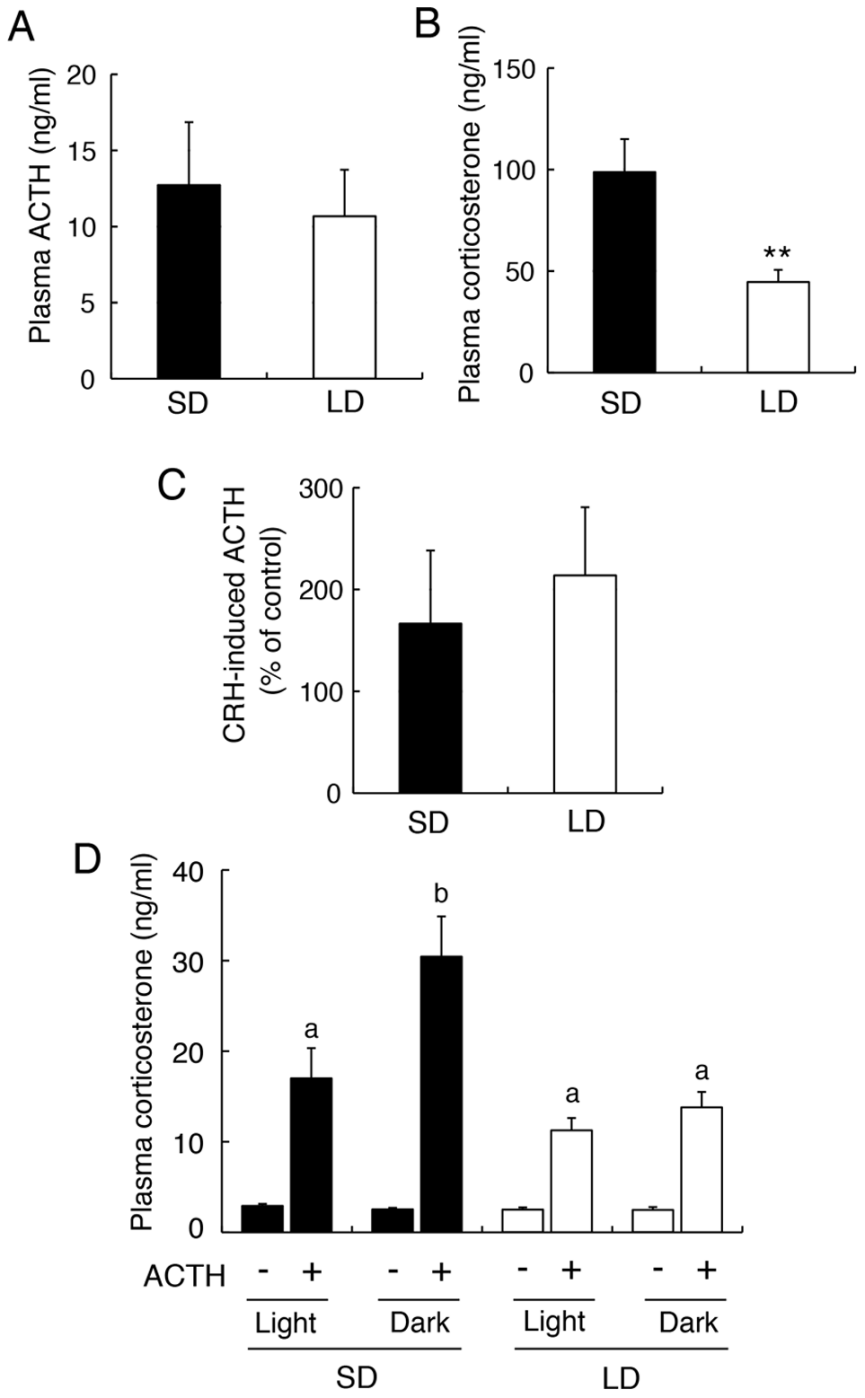
Effects of photoperiod on the HPA axis in Fischer 344 rats. Rats were maintained under either short-day condition (SD, 8 h of light, 16 h of darkness, *black bars*) or long-day condition (LD, 16 h of light, 8 h of darkness, *white bars*) for 2 weeks, and examined plasma concentrations of ACTH (A) and corticosterone (B) around Zeitgeber time 7–10 (ZT: ZT0 corresponds to the light onset). Corticosterone levels of rats under LD were significantly lower than those under SD (**, *P*<0.01, *t*-test). (C) CRH-induced ACTH secretion from the organ culture of hemi-pars distalis dissected from animals exposed to either SD or LD. The data are indicated as the percentage of the CRH-induced ACTH level to the control level for each rat (n=4–6). (D) ACTH-induced corticosterone levels in dexamethasone-treated rats maintained under SD and LD. Rats were intraperitoneally injected with dexamethasone-21-phosphate (0.97 μmol/kg body weight) 2 h prior to light onset (Light) or dark onset (Dark) under SD and LD, followed by intraperitoneal injections of rat ACTH_1–24_ (0.27 nmol/kg body weight) or saline 3 h after dexamethasone injections. Plasma samples were collected 20 min after ACTH injections. Different characters indicate significant differences within ACTH-injected groups (*P*<0.05, two-way ANOVA followed by Bonferroni's multiple comparison test). Values are means + SEM (n=3–7).

To elucidate the effects of photoperiod on hypophyseal sensitivity to CRH, we used organ cultures of the pars distalis dissected from rats exposed to long- and short-day conditions. The culture samples were prepared at ZT7–10 because corticosterone levels peaked around these times *in vivo* (Figure 2B). Plasma samples were collected from these animals to determine the concentrations of ACTH and corticosterone. ACTH levels did not change in response to the photoperiodic conditions (*P*>0.05) (Figure 3A), whereas corticosterone levels were lower in animals exposed to long-day condition compared to their levels in animals exposed to short-day condition (*P*<0.01) (Figure 3B). The CRH-induced ACTH level in the each pars distalis exceeds 100% of the respective control level except one sample of short-day condition, in which the CRH-induced level was 81% of the control. When the levels were compared between animals exposed to long- and short-day conditions, they did not differ significantly (*P*>0.05) (Figure 3C).

We further analyzed the sensitivity of the adrenal gland to ACTH by means of ACTH injections in dexamethasone-treated rats *in vivo*. The analysis was performed at light onset and dark onset under long- and short-day conditions. Dexamethasone injections suppressed plasma concentrations of corticosterone irrespective of injected time in rats exposed to long- and short-day conditions (Figure 3D). Intraperitoneal injections of ACTH in dexamethasone-treated rats significantly induced corticosterone levels under both photoperiodic conditions (F_1,30_=38.06, *P*<0.01, main effect of ACTH injections). When ACTH-induced levels were compared among ACTH-injected groups, they were significantly higher in rats exposed to short-day condition than in those exposed to long-day condition (F_1,16_=8.22, *P*<0.05, main effect of photoperiod). In rats exposed to short-day condition, the induced levels at dark onset were significantly higher than those at light onset (*P*<0.05), whereas no time differences were detected in rats exposed to long-day condition (*P*>0.05) (Figure 3D).

### Effects of Melatonin Injections on Corticosterone Rhythms

**Figure 4:**
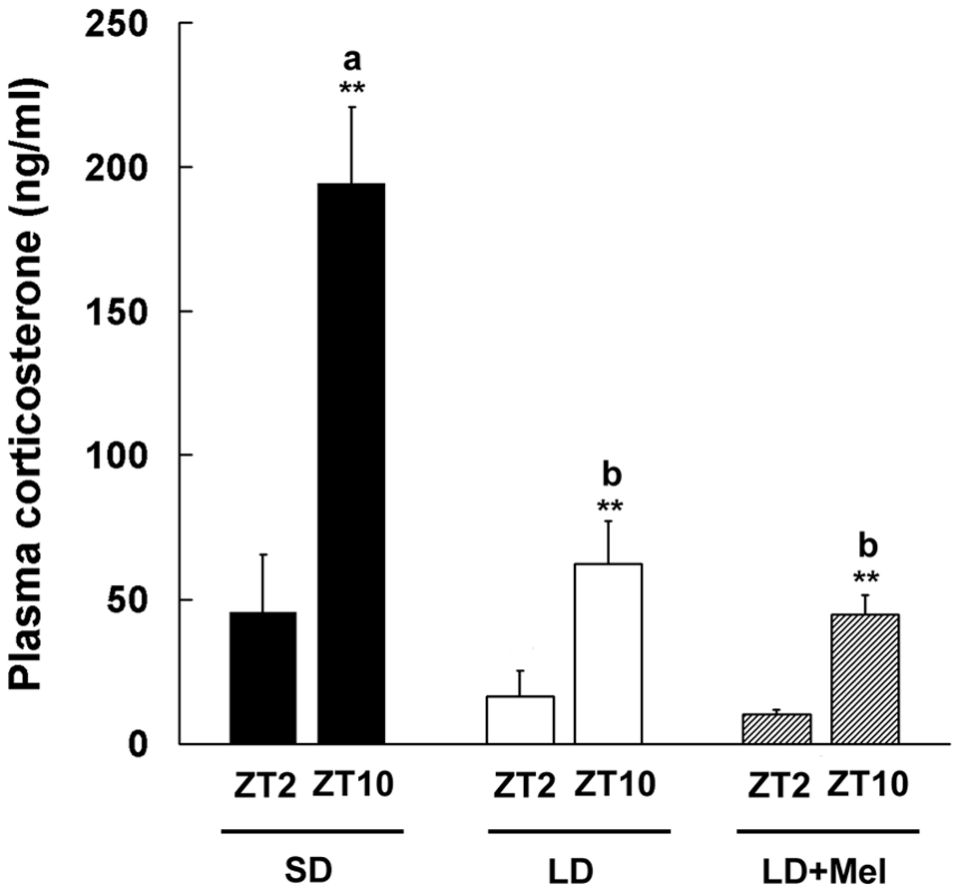
Effects of melatonin injections on plasma corticosterone rhythms in Fischer 344 rats. Rats maintained under long-day condition (LD, 16 h of light, 8 h of darkness) were daily injected either vehicle (*white bars*) or melatonin (*shaded bars*) at late afternoon for 3 weeks. The other group of rats was maintained under short-day condition (SD, 8 h of light, 16 h of darkness, *black bars*). Plasma concentrations of corticosterone at Zeitgeber time (ZT: ZT0 corresponds to the light onset) 2 and 10 are shown. Values are means + SEM (n=4–5). **, *P*<0.01, *t*-test. Different characters indicate significant differences among the levels at ZT10 (one-way ANOVA followed by Bonferroni's multiple comparison test, *P*<0.05).

To evaluate the involvement of melatonin signals in photoperiod-regulated corticosterone rhythms, rats were intraperitoneally injected with melatonin or vehicle at ZT14 under long-day condition for 3 weeks, and plasma corticosterone levels at ZT2 and 10 were compared to rats maintained under short-day condition. Corticosterone rhythms clearly amplified in rats maintained under short-day condition compared to those injected with vehicle under long-day condition (ZT10: F_2,9_=20.99, *P*<0.001) (Figure 4). However, melatonin injections did not mimic the effects of short-day condition on corticosterone rhythms, and the levels at both time points were similar with those in rats injected with vehicle under long-day condition (*P*>0.05) (Figure 4).

### Effects of Photoperiod on Corticosterone Rhythms and Adrenal Genes Expression in C57BL/6J Mice

**Figure 5:**
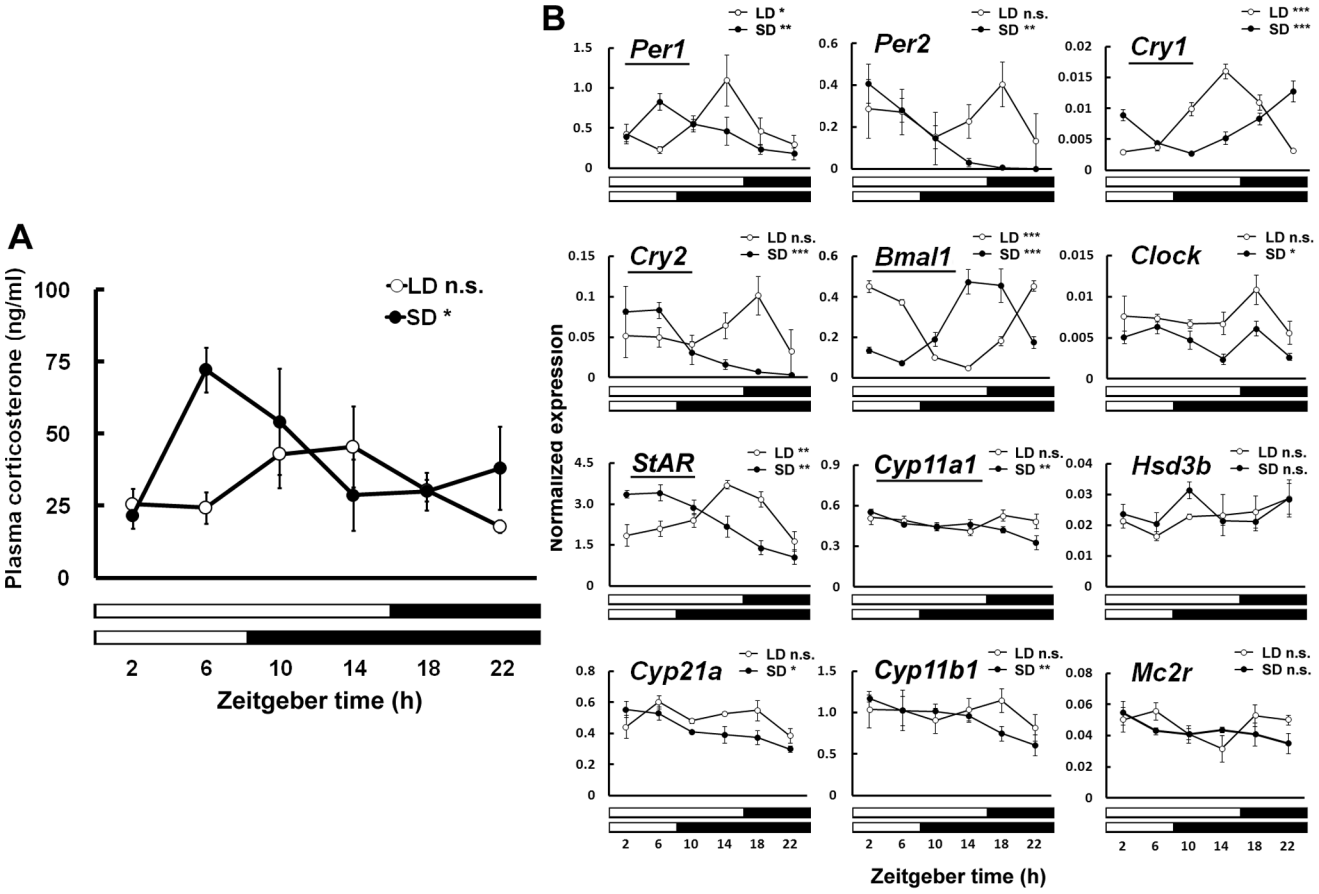
Effects of photoperiod on plasma corticosterone rhythms and expression of adrenal genes in C57BL/6J mice. Mice were maintained under either short-day condition (SD, 8 h of light, 16 h of darkness, *black circles*) or long-day condition (LD, 16 h of light, 8 h of darkness, *white circles*) for 2 weeks, and examined diurnal rhythms of plasma corticosterone (A) and expression of clock genes and steroidgenesis genes (B). Diurnal rhythmicity was analyzed by one-way ANOVA, and significances (*, *P*<0.05, **, *P*<0.01, ***, *P*<0.001) or non-significances (n.s.) were indicated in each graph. Significant effects of photoperiod on the expression profiles of adrenal genes were indicated by *underlines* of gene symbols (two-way ANOVA, interactions between photoperiod and time, *P*<0.05). Values are means ± SEM (n=4–10).

We further examined diurnal variations in plasma corticosterone levels in melatonin-deficient C57BL/6J mice exposed to long- and short-day conditions. Corticosterone levels exhibited a significant rhythm in mice exposed to short-day condition (F_5,26_=3.61, *P*<0.05) and peaked at ZT6 (Figure 5A), whereas no diurnal variation was detected in mice exposed to long-day condition (F_5,29_=1.91, *P*>0.05) (Figure 5A). Two-way ANOVA detected a significant interaction between photoperiod and time (F_5,55_=2.99, *P*<0.05).

Diurnal rhythms of expression of clock genes in the adrenal gland were analyzed in C57BL/6J maintained under long- and short-day conditions. All genes examined were expressed rhythmically in mice maintained under short-day condition (*Per1*: F_5,16_=5.09, *P*<0.01; *Per2*: F_5,16_=5.40, *P*<0.01; *Cry1*: F_5,16_=31.99, *P*<0.0001; *Cry2*: F_5,16_=7.52, *P*<0.001; *Bmal1*: F_5,16_=11.99, *P*<0.0001; *Clock*: F_5,16_=3.99, *P*<0.05). Several genes were also expressed rhythmically in mice maintained under long-day condition (*Per1*: F_5,14_=3.26, *P*<0.05; *Cry1*: F_5,14_=14.84, *P*<0.0001; *Bmal1*: F_5,14_=86.77, *P*<0.0001), although expression of *Per2, Cry2,* and *Clock* did not show significant rhythms (*P*>0.05) (Figure 5B). Two-way ANOVA detected significant interactions between photoperiod and time in the expression of *Per1* (F_5,30_=3.73, *P*<0.01), *Cry1* (F_5,30_=31.40, *P*<0.0001), *Cry2* (F_5,30_=2.70, *P*<0.01), and *Bmal1* (F_5,30_=32.01, *P*<0.0001), and the peak time points of the rhythms were either advanced 8 h (*Per1* and *Bmal1*) or delayed 8 h (*Cry1*) in mice maintained under short-day condition compared to those maintained under long-day condition (Figure 5B).

We also analyzed rhythmic expression of several genes involved in steroidgenesis, and found the significant rhythms of *StAR* (F_5,16_=10.83, *P*<0.0001), *Cyp11a1* (F_5,16_=5.37, *P*<0.01), *Cyp21a* (F_5,16_=4.91, *P*<0.01), and *Cyp11b1* (F_5,16_=3.07, *P*<0.05) in mice maintained under short-day condition (Figure 5B). On the other hand, only *StAR* was rhythmically expressed in mice maintained under long-day condition (F_5,14_=6.84, *P*<0.01)(Figure 5B). Significant interactions between photoperiod and time were detected in the expression of *StAR* (F_5,30_=11.18, *P*<0.0001) and *Cyp11a1* (F_5,30_=2.70, *P*<0.05). The peak of the rhythmic expression of *StAR* was 8 h advanced in mice maintained under short-day condition compared to those maintained under long-day condition (Figure 5B).

## Discussion

The mechanisms underlying photoperiodic regulation of stress physiology are poorly understood because of limited animal models that exhibit a clear response of their stress-related functions to photoperiodic stimuli. To establish a suitable model for this purpose, we here demonstrate for the first time the photoperiodic regulation in the HPA axis of Fischer 344 rats. These rats are known to exhibit robust responses to the photoperiod regarding their gonadal growth, body weight, and expression of key genes for the photoperiodic gonadal regulation in the brain [26], [27]. In agreement with these reports, we confirmed the greater body weight and epididymal fat mass in Fischer 344 rats exposed to long-day condition than those exposed to short-day condition. However, we could not detect a photoperiodic change in testicular weight. This discrepancy might be attributed to the stages in growth; testicular volume in young Fischer 344 rats (4–5 weeks old) exhibits a robust response to the photoperiod, which is reduced with age until reaching a point of nonresponse in adults despite the maintained sensitivity of body weight to the photoperiod during adulthood [26]. The rats in our study were 8–9 weeks old at the sampling day, and their testicular growth appears to have reached the nonresponsive point, whereas other physiological outputs, i.e., body weight and fat mass were sensitive to the photoperiod. Thus, these rats are suitable animals to detect the effects of photoperiod on HPA axis in the absence of gonadal changes.

Using the Fischer 344 rats, we first demonstrate the effects of photoperiod on diurnal variations in plasma concentrations of ACTH and corticosterone. Surprisingly, temporal patterns of ACTH levels did not exhibit positive correlations with those of corticosterone levels; ACTH levels had no fluctuations over 24 h under long- and short-day conditions, whereas corticosterone levels exhibited a robust rhythm under short-day condition that was attenuated under long-day condition. These data are inconsistent with the classical concept that plasma concentrations of ACTH and corticosterone are positively linked and exhibit parallel rhythms under 12L:12D in many mammalian species including rats [30] and mice [24]. However, a corticosterone rhythm persists in hypophysectomized rats implanted with ACTH pellets under 12L:12D [31], presumably through diurnal changes in adrenal sensitivity to ACTH [14], [15]. These data raise the hypothesis that photoperiod affects the sensitivity of adrenal gland regarding its regulation of corticosterone rhythms. In agreement with this hypothesis, under the dexamethasone-treated condition *in vivo*, i.e., the condition in which endogenous ACTH is suppressed, the sensitivity of adrenal corticosterone secretion to injected ACTH was higher in rats exposed to short-day condition than in those exposed to long-day condition in the present study. Notably, the sensitivity increased at dark onset under short-day condition, at which corticosterone rhythms reached their peaks. These data strongly suggest that a short photoperiod sensitizes the adrenal gland to ACTH to generate a robust corticosterone rhythm. This hypothesis is further supported by a report using fat sand rats captured from Algerian Sahara desert, in which the sensitivity of cortisol secretion to ACTH increases during winter and decreases during spring [32].

Although melatonin is widely known as a transmitter of the photoperiodic information in mammals [16], [17], a daily melatonin injection at late afternoon under long-day condition did not induce the amplified rhythm observed under short-day condition in the present study. In addition, melatonin-deficient C57BL mice also showed a clear rhythm under short-day condition, which was attenuated by the exposure to long-day condition. These data suggest that melatonin is not crucial for the photoperiodic regulation of corticosterone rhythms. In mammals, significant evidence suggests the involvement of SCN-autonomic innervations in the circadian rhythms of glucocorticoid secretion [12], [33], as lesions of the SCN abolish the diurnal rhythms of corticosterone [34] and adrenal sensitivity [35], which are reduced in SCN-intact, adrenal-denervated animals [36]. Additionally, light induces a variety of genes including clock genes *Per1* and *Per2* in the adrenal gland with corticosterone release via SCN-sympathetic nervous system in mice [23]. Thus, our data on 1) photoperiod-regulated adrenal sensitivity in rats, and 2) melatonin-independent responses of corticosterone rhythms to photoperiod in rats and mice, suggest that photoperiod affects corticosterone rhythms via an autonomic pathway. This is also inferred by the photoperiodic regulation of immune functions by the sympathoadrenal system in Siberian hamsters [22]. Notably, in Syrian hamsters, glucocorticoid rhythms regulate the nycthemeral and photoperiodic changes in tryptophan hydroxylase 2 gene, which codes the rate-limiting enzyme of serotonin synthesis, in the raphe nuclei [37]. Since serotonin is one of the major regulators of the master clock in the SCN, and the SCN receives direct serotonergic innervations from the median raphe as well as an indirect input from the dorsal raphe [38], a glucocorticoid rhythm itself may modulate/enhance the output from the SCN such as adrenal sensitivity via the serotonergic system.

Rhythmically expressed clock genes in the adrenal gland play pivotal roles in generating corticosterone rhythms in mice under 12L:12D or constant darkness, as shown by using *Per2*/*Cry1* double mutant mice [24] or adrenal-specific knockdown of BMAL1 protein [25]. In these lighting conditions, *StAR* and other genes involved in corticosterone biosynthesis are controlled by the adrenal clock [24], [25], [39]. Here we demonstrate the temporal expression of clock genes and several genes involved in steroidgenesis under different photoperiodic conditions in mice. Intriguingly, rhythmic expression of several clock genes and *StAR* were 8 h advanced under short-day condition compared to long-day condition, correlated with the 8 h advance of light-dark transition under short-day condition relative to that under long-day condition. An exceptional case is *Cry1* expression, whose phase was 8 h delayed under short-day condition. The ACTH receptor gene, *Mc2r*, did not show diurnal or photoperiodic changes, although a previous report showed the circadian rhythm of the *Mc2r* expression in mice adrenal gland under constant darkness [24]. Exposure to light irrespective of photoperiod might modify the expression, as *Mc2r* expression is sensitive to a light pulse in the adrenal gland of rats [40]. Other important observation is that short-photoperiod induced significant rhythmicity of 10 genes/12 genes examined, whereas only several genes among them (4 genes/12 genes) showed significant rhythmicity under long-day condition. These observations suggest that photoperiod differentially entrains the adrenal clock and its downstream genes involved in steroidgenesis. However, photoperiod did not alter the amplitude of the rhythms of adrenal genes examined despite of the amplified corticosterone rhythms in plasma under short-day condition. Genome-wide studies are needed to identify the genes responsible for the amplification of corticosterone rhythms in response to photoperiod.

In conclusion, photoperiod regulates plasma corticosterone rhythms in Fischer 344 rats through the adrenal sensitivity to ACTH. Photoperiodic changes in corticosterone rhythms are likely to be controlled by melatonin-independent mechanisms, as demonstrated using melatonin injected rats and melatonin-deficient C57BL/6J mice. Our study contributes to understanding the mechanisms underlying seasonal regulation in stress-related physiology as well as seasonal affective disorder, a disease characterized as a condition of regularly occurring depression in winter and at least in part regulated by seasonal changes in HPA axis.

## Materials and Methods

### Ethics Statement

All animal experiments reported here were conducted in accordance with the Guidelines for Animal Experiments of the Faculty of Agriculture of Kyushu University, as well as the Law (No. 105) and Notification (No. 6) of the Japanese Government, and were specifically approved by Animal Care and Use Committee of Kyushu University.

### Animals

Male 4-week-old Fischer 344 rats and C57BL/6J mice were obtained from Charles River Laboratories (Yokohama, Japan) and Japan SLC (Shizuoka, Japan), respectively. They were maintained in light-tight boxes and exposed to short-day condition [8 h of light (50 lux), 16 h of darkness (8L:16D)] for at least 1 week before the experiment. Five to 6-week-old rats or mice were separated into 2 groups: 1 group was transferred to the long-day condition by delaying the lights-off time by 8 h (16L:8D) for 2 weeks and the other group was maintained under short-day condition for additional 2 weeks. Thereafter, they were used for either plasma collections for hormonal assays, organ cultures, ACTH injections, and mRNA analysis in adrenal gland as described below. The animal boxes were placed in a room at a temperature of 25±1°C. Standard diet for laboratory rodents (MF, Oriental Yeast, Tokyo, Japan) and water were available *ad libitum*. Although the previous report used a low-calorie diet (ZF for herbivorous animals, Oriental Yeast) to enhance the photoperiodic response of gonads in Fischer 344 rats [27], our pilot experiments revealed no enhancement of the stress response to the photoperiod by the use of ZF (data not shown). Rather, the ZF diet appears to behave as an overall stressor irrespective of photoperiodic conditions and may mask the photoperiod-specific effects. Thus, we used standard MF diet throughout the present study.

### Diurnal Rhythm of Plasma Hormones and Gene Expression in Adrenal Gland

Rats and mice were sacrificed by isoflurane anesthesia and decapitated within 2 min to avoid acute increases in corticosterone levels, and trunk blood was collected into sampling tubes. They were sacrificed at ZT2, 6, 10, 14, 18, and 22 (n=4–5). Decapitation during the dark phase was performed under dim red light. In rats, body mass, testes, and epididymal fat were weighed to confirm the photoperiodic response. In mice, adrenal glands were collected in RNA*later* (Ambion, TX, USA). Blood was immediately chilled after the collection and centrifuged at 3000 rpm for 10 min at 4°C, and collected plasma samples were stored at −80°C until analysis. Highly hemolyzed plasma samples were removed from the analysis, since they influence the stability of ACTH.

### Organ Culture

Culture preparation was performed at ZT7–10. Rats were sacrificed by isoflurane anesthesia, and the hypophysis was dissected from the sella turcica. The pars nervosa was carefully removed under dissection microscopy, and then the pars distalis was divided into 2 equal pieces. Each hemi-pars distalis was cultured on the culture inserts (BD Falcon, Franklin Lakes, NJ) with 1 ml of DMEM (Invitrogen, Carlsbad, CA) supplemented with 10 mM HEPES, 25 U/ml penicillin, 25 mg/mg streptomycin, and 2% B27 supplement (Invitrogen) at 37°C under 95% atmosphere and 5% CO_2_ for 24 h. Thereafter, the medium was replaced with prewarmed medium including either 100 nM CRH (Peptide Institute, Osaka, Japan) or vehicle. Each hemi-pars distalis from the same rat was treated with either CRH or vehicle (n=4–5). After 3 h of stimulation, medium was collected for ACTH measurement and stored at −80°C until analysis. After the data were normalized by individual tissue weight, they were calculated according to the percentage of the CRH-induced value to the control value for each animal.

### ACTH Injections

ACTH injections were performed according to a previously reported method [41]. Rats were intraperitoneally injected with dexamethasone-21-phosphate (0.97 μmol/kg body weight, Sigma, St. Louis, MO) 2 h prior to light onset or dark onset under short- and long-day conditions (ZT22 or 6 under short-day condition, ZT22 or 14 under long-day condition) and left in their home cages for 3 h. Thereafter, they were intraperitoneally injected with rat ACTH_1–24_ (0.27 nmol/kg body weight, Sigma) or vehicle (0.9% NaCl), left an additional 20 min in their home cages, and rapidly decapitated under isoflurane anesthesia (n=3–7). Samples were stored at −80°C until the assay. The injected dose of dexamethasone was sufficient to suppress endogenous ACTH, and that of ACTH yielded circulating levels of ACTH that were within the physiological range in Fischer 344 rats [41].

### Melatonin Injections

The melatonin injections were performed according to a previous study [27]. Four-week-old Fischer 344 rats were obtained from Charles River Laboratories, and maintained under short-day condition for 1 week. At 5-week-old, rats were separated into 3 groups (n=8–9 per group): one group was maintained under short-day condition, and the other two groups were transferred to long-day condition. Three weeks after the separation, a daily melatonin injection was started with the groups maintained under long-day condition, i.e., they were intraperitoneally injected with either melatonin (100 µg of melatonin dissolved in 0.1 ml 10% ethanol and 0.9% NaCl) or vehicle (0.1 ml 10% ethanol and 0.9% NaCl) 2 h prior to the dark onset (ZT14). Injections were carried out for 3 weeks. Rats maintained under short-day condition were left intact. Thereafter, rats were sacrificed by decapitation under isoflurane anesthesia within 2 min at ZT 2 and 10 (n=4–5 per time point for each group). Blood was centrifuged at 3000 rpm for 10 min at 4°C, and collected plasma samples were stored at −80°C until analysis.

### Hormone Assays

Total corticosterone was measured in duplicate by using a corticosterone enzyme immunoassay kit (Cayman Chemical, Ann Arbor, MI) according to the manufacturer's protocol except for the use of Steroid Displacement Reagent (2.5%, Enzo Life Sciences, Farmingdale, NY) in the step of plasma dilution. The cross-reactivities of the antibody are following: corticosterone, 100%; 11-dehydrocorticosterone, 11%; 11-deoxycorticosterone, 7%; progesterone, 0.31%; cortisol, 0.17%; aldosterone, 0.06%. Intra- and inter-assay coefficients of variation were 2.8% and 8.9%, respectively.

Plasma ACTH concentrations were measured by RIA as described by Katoh et al. [42]. A specific antiserum was kindly provided by Dr. B.J. Canny (Monash University, Australia) and shows following cross-reactivities: ACTH_1–39_, 100%; ACTH_5–24_, 1.6%; ACTH_18–39_, <0.01%; β-endorphin_1–31_, <0.01%; α-melanocyte-stimulating hormone, <0.01%; γ-melanocyte-stimulating hormone, <0.01%; and deacetyl-α-melanocyte-stimulating hormone, <0.01%. Intra- and inter-assay coefficients of variation were 9.8% and 12.4%, respectively.

### Real-time PCR

Total RNA was isolated using ISOGEN (Nippon gene, Tokyo, Japan) from whole adrenal gland, and cDNA was synthesized using the PrimeScript RT Reagent Kit with gDNA Eraser (Takara, Ohtsu, Japan) according to the manufacturer's protocol. In order to analyze the expression levels of clock genes (*Per1*, *Per2*, *Cry1*, *Cry2*, *Bmal1*, and *Clock*), genes involved in steroidgenesis (*StAR, Cyp11a1, Hsd3b, Cyp21a,* and *Cyp11b1*), and the ACTH receptor gene (*Mc2r*), real-time PCR was performed using Mx3000P Real–Time QPCR System (Stratagene, CA, USA) with SYBR *Premix Ex Taq* (Takara) and primers shown in Table 1. PCR was performed at 95°C for 30 s followed by 40 cycles at 95°C for 5 s, 60°C for 30 s. The specificity of PCR products was confirmed by analyzing the dissociation curves. Levels of *cyclophillin* mRNA were used as internal controls.

**Table 1:** Primers used for real-time PCR

gene		5′–3′ sequence	size
*Per1*	F	agaagaaaacagcaccagct	98
	R	tcttgagttataagaaccccaacatg	
*Per2*	F	gccaagtttgtggagttcctg	226
	R	cttgcaccttgaccaggtagg	
*Cry1*	F	gtcattgcaggaaaatgggaag	235
	R	taaagaggcggagagacaaagg	
*Cry2*	F	agatggcctcaggttttctcag	218
	R	ttcaggcccactctaccttctc	
*Clock*	F	gtggtgactgcctatcctacct	286
	R	aaggagggaaagtgctctgttg	
*Bmal1*	F	gcagtgccactgactaccaaga	170
	R	tcctggacattgcattgcat	
*StAR*	F	ttgggcatactcaacaacca	102
	R	gaaacaccttgcccacatct	
*Cyp11a1*	F	gacctggaaggaccatgca	63
	R	tgggtgtactcatcagctttattga	
*Hsd3b*	F	agaccagaaaccagggagcaa	84
	R	tctccttccaacactgtcacctt	
*Cyp21a*	F	gggaactgcccagcaagtt	78
	R	ggatggtgttctgggattcttc	
*Cyp11b1*	F	tcagtccagtgtgttcaactatacca	62
	R	gccgctccccaaaaaga	
*Mc2r*	F	cacaaatgattctgctgcttcc	188
	R	ttatttcttgcggtgtcattgg	
*Cyclophillin*	F	cgactccggcaagatcgaa	67
	R	ggtcccccaggctctctact	

### Statistical Analysis

Student's *t*-test was used for the analysis of the photoperiodic effects on body weight, epididymal fat mass, testicular weight, and hormonal levels in plasma or culture medium at a single time point. Diurnal variations in plasma ACTH, plasma corticosterone, and adrenal genes expression were analyzed by one-way ANOVA, followed by Bonferroni's multiple comparison test. Two-way ANOVA was applied for the analysis of the effects of photoperiod on diurnal variations in hormonal levels and adrenal genes expression, as well as effects of ACTH or melatonin injections on corticosterone levels. Adrenal sensitivity to ACTH was further analyzed by one-way ANOVA within ACTH-injected groups followed by Bonferroni's multiple comparison test. Values were considered significantly different at *P* < 0.05.
